# Exploring chiropractic students’ experiences of the educational environment in healthcare professional training: a qualitative study

**DOI:** 10.1186/s12909-015-0417-z

**Published:** 2015-08-05

**Authors:** Per J. Palmgren, Klara Bolander Laksov

**Affiliations:** 1 Department of Learning, Informatics, Management and Ethics, Karolinska Institutet, Stockholm, 171 77 Sweden; 2 Department of Education, Stockholm University, Stockholm, Sweden

## Abstract

**Background:**

The educational environment has a significant impact on students’ behavior, sense of well-being, and academic advancement. While various research methodologies have been used to explore the educational environment, there is a paucity of studies employing qualitative research methods. This study engages in an in-depth exploration of chiropractic students’ experiences of the meaning of the educational environment.

**Methods:**

A qualitative approach was employed by interviewing 26 students in four focus group interviews at two different points in time. A conventional manifest and latent content analysis was chosen to investigate and interpret the experiences of the educational environment in an undergraduate chiropractic training institution in Sweden.

**Results:**

The analysis resulted in five overarching themes: Personal growth; Being part of a community; A place of meaningfulness; Trust in a regulated system; and Scaffolding relationships. Early in the training, the meaning of the educational environment was experienced as part of a vocational community and the scaffolding of intra-institutional relationships. In later stages, the environment was experienced in terms of personal growth – balancing academic pressures and progress within the professional community – thus laying the foundations for autonomy and motivation. During the clinical training, the environment was experienced as where learning happens, thus creating a place of meaningfulness. Throughout the training, the formal and clinical environments were experienced as isolating, with little bridging between the two. A regulated system – conveying an operative organization with clear communication regarding what to expect – was experienced as important for an apt educational environment.

**Conclusions:**

We found that experiences of an educational environment are dynamic and change over time. When restructuring or evaluating curriculums, educational managers can consider the emerged themes as constituting facets relating to the educational environment, and thus possible learning conditions. Likewise, researchers can consider these aspects of the educational environment when: interpreting results from quantitative and qualitative inquiries, constructing and refining instruments, or conceptualizing and framing the educational environment phenomenon.

## Background

Healthcare professional training environments have been increasingly acknowledged as imperative for high-quality education [1, 2]. These environments evolve in conjunction with teaching and learning, can be both academic and clinical, and occur in both formal and informal encounters. Exploring educational environments can be intricate as they encompass many features and settings [3]. They can be viewed as interactions between groups of people with a vested interest and their organizational structure where students are one of the key stakeholders.

In his seminal papers, Genn [4, 5] details a vivid discourse on the concepts around the educational environment and asserts that students’ perceptions of the environment are related to their achievements, satisfaction, and success. This notion has been further supported with empirical investigations and underpinned with research outcomes [5–7]. Moreover, scholarly work has shown that organizational changes impact educational environments [4, 8] and that dysfunctional environments are costly and counter-productive [9].

The phenomenon of the educational environment, variously synonymized as spirit, climate, or culture, is complex and multifaceted [10]. Although frequently used in different forms, the concept is rarely well-defined, and a clear definition remains elusive. One plausible reason for this deficiency of conceptual framing could be that researchers were initially more occupied with attempting to measure the concept rather than trying to conceptualize and theorize it [11]. However, for the purpose of this study, we were inspired by a comprehensive operational definition of the phenomenon from the standpoint of organizational research. This perceives the environment as a broad concept, potentially including all internal and external organizationally-related phenomena, with climate and culture describing subsets of the internal environment [12]. While the existing literature describes the impact and importance of educational environments, relatively little research has explored the constituents of such environments. Although the word “environment” is synonymous with physical space, it also has social, emotional, and intellectual connotations, and the use of the concept with its all-embracing nature has been criticized [3]. Explorations of the phenomenon began in the 1930s, and hastened with the work of Pace and Stern [13] and Moos [14], with a curiosity about educational institutions as social organizations and structures.

Several research approaches have been employed to explore and understand the somewhat ethereal features of educational environments, including qualitative [15–17], quantitative [18–20], and mixed-methods [21, 22] approaches. Various instruments can be used to measure educational environments in professional healthcare education, each with its strengths and drawbacks. The Dundee Ready Educational Environment Measure (DREEM) is undoubtedly the most extensively utilized instrument [1, 23] and gauges the undergraduate educational environment with items allocated to five subscales of direct relevance to the concept.

The use of instruments can be intricate and multifarious because of the risk of excluding central elements. Quantitative introspections can provide useful information about students’ perceptions, but they offer restricted acumen into the intricacy of educational environments [24]. Scholarly works from inventory-based investigations often conclude with calls for qualitative explorations and inductive approaches to generate a deeper understanding of the context and concept. Even though DREEM findings have been compared with interviews [22], we have yet to identify studies following up quantitative results with qualitative explorations to further explore the phenomenon, thus suggesting a gap in the scientific literature. There is also a paucity of empirical investigations on changes in the educational environment over time, and existing studies are primarily quantitative in nature [8, 25, 26].

Schönrock et al. [11] report that many measures are not grounded in theory. They consequently propose a theoretical framework based on a literature review and empirical investigations developed by Moos [14] to underpin investigations of the phenomenon. According to Moos [14], human environments can be conceptualized within three broad domains: personal development or goal direction; relationship; and system maintenance and system change. Moos persuasively argues that the aforementioned domains underpin most socially created environments and that vastly different social environments, including educational, can be investigated using these social domains. Moreover, given the limited psychometric evidence relating to scores from existing tools to assess educational environments, and the pledge of refining prevailing and/or constructing new tools [23], inductive approaches and naturalistic inquiry can be used complementarily to generate models to scaffold the phenomenon under study.

Several professions, such as medicine, dentistry, and nursing, have done a great degree of empirical introspection, both quantitative and qualitative, regarding the educational environment [8, 16, 27–31]. However, despite some survey-based investigations among chiropractic students in Canada [6, 32] and Sweden [26, 33], to our knowledge, there are no existing qualitative explorations of how this group experiences the educational environment. Furthermore, our earlier cross-sectional and longitudinal instrument-driven investigations [26, 33] motivated us to again investigate this group, employing another methodological approach to more fully comprehend the phenomenon of the educational environment. Generally, therefore, there is a dearth of research employing qualitative methods and methodologies beyond those of the post-positivist paradigm, and in-depth qualitative explorations focusing on the temporal course are warranted.

We did not explicitly set out to examine learning connected to the environment but rather to identify conditions for learning and apprehend the intangible construct and diversified facets of the environment within which education is delivered. However, as teaching and learning constitute a major part of education, and in order to lever and transfer findings to a more general level, sociocultural frameworks, like communities of practice, can help broaden the perspective [11]. Characteristic of sociocultural theories is that interaction and collaboration with others are acknowledged as influencing students’ learning processes as they become familiar with the norms and attitudes in the communities to which they are being introduced.

This study sought an in-depth exploration of undergraduate chiropractic students’ experiences of the meaning of their educational environment. Students’ perspectives can generate a greater understanding of the characteristics and conditions for learning constituting the educational environment and how these are experienced over time. Thus, the specific research questions were: How is the educational environment experienced at different points in time? Which learning conditions are experienced as constituting facets of the educational environment?

## Methods

### Context

This study was conducted at a chiropractic college in Sweden, the Scandinavian College of Chiropractic (SCC), a university college offering a 5-year full-time undergraduate program in chiropractic. The program is divided into a conventional preclinical phase and a 2-year clinical phase. The former focuses on basic and clinical sciences and theoretical and practical training in traditional and formal classroom settings in the first 3 years, including early clinical placements for a few days each semester. The latter takes place at the institution’s outpatient clinic and is interwoven with formal theoretical education. After graduation and a 1-year internship in public healthcare, the National Board of Health and Welfare issues a professional status qualification in chiropractic (Registered Chiropractor).

### Study design and methodology

The study was part of a larger project employing a prospective mixed-methods multiple case study methodology anchored in a pragmatic research tradition, as outlined by Creswell [34].

To explore experiences, a qualitative, interpretive approach was chosen, thus examining the phenomenon in its natural setting [34], with the assumption that knowledge is situated and socially constructed. The findings in this paper are viewed as being shaped during interaction between the study participants and the investigators [35]. They do not mirror a strictly objective truth and are seen as transferable to other contexts [36].

The study was informed by communities of practice, a framework described by Wenger [37] as “Groups of people who share a concern or passion for something they do and learn how to do it better as they interact regularly.” Noteworthy, this framework inadvertently allows for processes of social learning. Thus, learning can be, and often is, an ancillary outcome that conveys these social processes, and the educational environment is reliant on these processes. We were inspired by communities of practice in framing the phenomenon under study and used it as a bifocal to better understand our findings.

### Participants

Four focus group interviews were conducted as part of a larger project, two during the spring terms of 2009 and 2012, respectively, with undergraduate students from years 1 and 4 interviewed separately. Twenty-six students participated, 12 women and 14 men, aged between 18 and 29 years. One group of participants (*n* = 6), three women and three men, comprised the same group during the study and were interviewed at two different time points – in 2009 as year 1 students and in 2012 as year 4 students – assembling a somewhat longitudinal sample. The incentive for the qualitative exploration of these cohort years was derived from empirical findings suggesting that students tend to be more optimistic about their environment early in their training while those who have surpassed the midpoint of their education often have less optimistic attitudes [6, 26, 33]. To achieve variation and breadth in the data, a purposeful criterion sample was chosen [38] based on the students’ gender, year of class and year (time point). Students were approached by mail, and all except one who declined due to time constraints accepted the invitation to participate. The selected number of participants was within the acceptable norm for effective and meaningful focus group discussions [39, 40].

### Data collection

Focus group interviews were used to promote interaction, different viewpoints, and dialogue [41]. An interview guide was developed to navigate discussions and mainly included questions relating to the five DREEM subscales – students’ perceptions of learning, teaching, academic self-perceptions, atmosphere, and social self-perceptions – which were considered germane. A pilot interview was conducted to test the interview guide [38]. Adopting the method outlined by Krueger [39], the overall interview structure was compiled using a series of carefully planned, introductory, transitional, key, and ending questions phrased in a conversational manner. The audio-taped group sessions were transcribed verbatim by someone independent from the study. The principal investigator served as the moderator, and interviews were conducted in a meeting room at the SCC, each lasting 75–90 mins.

### Data analysis

**Fig. 1:**
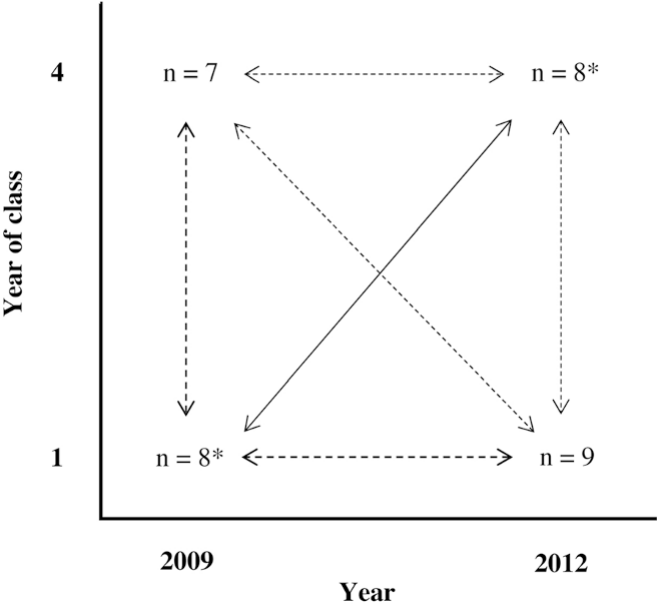
Cross-contrasting groups. The figure depicts the analytical process of cross-contrasting sub-categories and categories in the four focus groups comprising the 26 participating students. *One group of participants (*n* = 6) comprised was interviewed at two different time points

An inductive qualitative content analysis was employed to explore the data [42, 43]. The transcripts were examined line-by-line, and sub-categories and categories were developed without predetermined coding schemes. The analysis incorporated several steps: i) the transcribed interviews were read numerous times by PJP to become familiar with the text and to identify meaning units relating to the aim of the study and the questions in the interview guide; ii) the meaning units were condensed, and codes depicting the phenomenon under investigation were created by PJP and KBL; iii) the codes were unitized and abstracted into sub-categories and categories describing the manifest content of the data and were iteratively discussed by both authors; iv) interpretative cross-contrasting of sub-categories and categories were performed (Fig. 1); and v) the analysis focused primarily on an interpretational level, i.e., the investigators went beyond the explicit manifest content. Sub-categories and categories were interpreted and explored into themes expressing the underlying latent content of the data [42]. Thus, the qualitative analysis pertain to a communication theory as described by Watzlawick et al. [35], suggesting a depiction of the manifest content as what the text explicitly says and the latent content as what the text implicitly talks about and the underlying meanings. Although the steps above seem sequentially ordered, the analytical process and search for patterns was in no way linear; rather, it was dynamic, iterative, and recursive. While performing this ingeminated analysis, it became apparent that some categories were somewhat congruent with the model of human environment proposed by Moos [14]. Therefore, in the latter stage of the data analysis, this framework was juxtaposed with the emerging data and used as a lens for further analysis. Noteworthy, however, the analysis was still inductive in nature. During this analytical phase, another investigator (a senior researcher not eligible as author) was recruited and contributed to the investigative process. The issue of methodological rigor was variously addressed. The trustworthiness of the analysis was enhanced by investigator (with different professional backgrounds) triangulation. Throughout the analytical process, and primarily due to the principal investigator’s prior understanding of the empirical context, constant comparisons between the sub-categories and categories and the original data transcripts were made to ensure a good fit between the data and findings. We thus gave careful consideration to Patton’s dual criteria of internal homogeneity and external heterogeneity [38]. Emerging themes were continually discussed until a consensus was reached among the investigators.

### Ethical considerations

Information about the study was sent via e-mail to students who had agreed to participate. They were then further informed about the study orally and in writing. Participation was voluntary, and the students were informed that they could withdraw at any time. Written informed consent was obtained from the participants prior to the interviews, and full confidentiality was guaranteed. None of the information collected was identifiable, thus ensuring data anonymity. The study was conducted according to the tenets of the World Medical Association Declaration of Helsinki and approved by the Regional Ethical Review Board in Stockholm (2012/416-31/5).

## Results

**Fig. 2:**
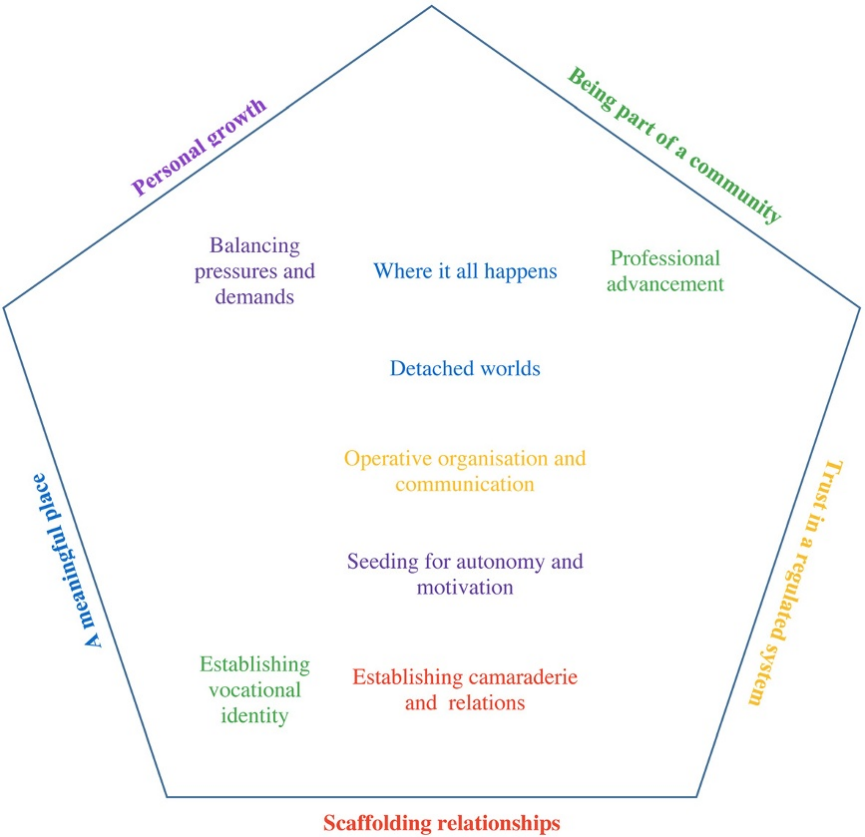
Students’ experiences of the meaning of the educational environment. An illustration of the emerging five latent themes, with manifest categories arranged vertically (*class year*) and horizontally (*time point*). The categories in the center column emerged from the four interviews (regardless of class year or time point) and connected and underpinned the realms of preclinical and clinical education, organizational and communicative issues, and training to encourage independence and aspiration. In the early years, it was categorically about developing an identity and creating bonds; in later years, it was about managing workload and burden, the meaningfulness of clinical education, and becoming a professional. During both training points (*longitudinal*), the students stated that belonging to a chiropractic community was paramount for a sound educational environment. By belonging to a community with an established identity, the students were offered professional prosperity within a solid structural and functional organization with clear and anticipatory communication

The analysis resulted in five overarching themes describing the students’ experiences of the meaning of the educational environment: *Personal growth; Being part of a community; A place of meaningfulness; Trust in a regulated system;* and *Scaffolding relationships* (Table 1). Each theme is presented using the underlying categories and illustrated with supporting quotes. The reciprocal connections between the categories and their emerging and encompassing within-case themes are illustrated in Fig. 2.

**Table 1:** The experienced educational environment: scheme of sub-categories, categories, and themes

Subcategory	Category	Theme
Different stressors	Balancing pressures and demands	Personal growth
Need for support when stressed		
The guide by the side	Seeding for autonomy and motivation	
Becoming self-directed		
Individual process of learning		
Defining the profession	Establishing vocational identity	Being part of a community
Vocation-related learning	Professional advancement	
Learning during internship	Where it all happens	A place of meaningfulness
“The penny drops on the clinic”		
Barrier between preclinical and clinical training	Detached worlds	
Flow from systematic to fragmentary teaching		
Forward planning	Operative organisation and communication	Trust in a regulated system
Importance of the physical environment		
Clear communication and information		
Feeling of smallness		
Feeling of closeness	Establishing camaraderie and relations	Scaffolding relationships
Social integration and interaction		
A feeling of egalitarianism and equity

### Personal growth

This theme was interpreted within two categories: *Balancing pressures and demands* and *Seeding for autonomy and motivation.*

The students experienced different types of stress, pressures, and demands. When demands came from the institution and peers and were moderate in nature, the will to perform increased. In order to grow personally and professionally, the students experienced demands as positive, motivating, and developmental as long as these did not transcend into negative pressure and stress, which became counter-productive.

*…you need the demands, but you also need some stress in order to learn, or at least I do. I think there are […] different kinds of stress – in the beginning, you had many lectures and lab work. Now, there is mainly the clinical phase integrated with many small clinical courses. This can put stress on you as a student to have the discipline to revise and rehearse all the time. It is all about balancing, but I guess coping with this stress is also part of our progression and development*. (Female, Year 4, 2009)

When students felt deprived or stressed, their support mechanisms were considered important aspects of a good institutional environment. However, there was a lack of awareness of existing formal support systems during stressful periods.

*…I have never been in that kind of situation, but I knew about the support mechanism because I was involved in the students’ union […] I think many students don’t know about it. Personally, I think I would first turn to a friend or maybe a teacher if I had problems or felt bad because you need to ventilate things; how can you develop otherwise?* (Female, Year 4, 2009)

Motivation was experienced as the antipole of pressures and excessive demands, thus acting as their counterweight in a successful educational environment. Students’ motivation to learn and study was significantly influenced by inspiring, well-prepared teachers who encouraged self-direction. However, this seeding for motivation and engagement was less marked during the clinical period than in the preclinical training period.

*It feels like it’s very much up to you as an individual, what you should know, and what you have learned. One has to be disciplined and perhaps look up information or talk to a teacher or peer. It’s really good to feel independent […] but I think that teachers should stimulate you to become that way; this is not always the case.* (Female, Year 4, 2012)

The students experienced the environment as preparing them sufficiently for working life, i.e., being self-directed and taking responsibility and initiative. This level of autonomy became more apparent as the training advanced but was experienced as more stochastically than pedagogically induced.

*…I find it hard to imagine that anyone who completes a 5-year education feels ready to go out to work. I don’t think it is possible to prepare anybody for working in that way; but it feels like at least I know what tools I should use now.* (Male, Year 4, 2012)

While motivation generated educational propulsion, stress was experienced as easily keeling this, and teachers’ pedagogical skills were seen as fundamental in promoting personal growth.

### Being part of a community

Two categories shaped the interpretation of this theme: *Establishing vocational identity* and *Professional advancement.*

The students saw themselves becoming part of a community to which they did not previously belong. A sound educational environment depended on assuming a chiropractic identity early on and knowing the profession. They considered prompt professional integration and the establishment of peer and mentor role models important in understanding, appreciating, and justifying their career path. Further, they conveyed feelings of being distanced from other health professional trainings and that a nurturing environment that was sensitive to their future inter-professional role within the healthcare system was important.

*I feel I’m beginning to understand what chiropractic is, and I’m grasping… chiropractic thinking… but I’d like to become more involved in the professional thinking even though I’ve only been in school for nine months. I hope we’ll be initiated in the manner of working and what’s expected of us as future chiropractors by working in teams with medical doctors, physiotherapists, or psychologists.* (Male, Year 1, 2009)

The students considered vocation-specific learning and the relevance of subjects to the profession as important cornerstones of an all-encompassing educational environment. In order to grow professionally and approach the chiropractic community, there was a sense of wanting to grasp “the tricks of the trade” and an urgency to establish an apprenticeship model.

*…one idea is that we could have one or two days a week with the chiropractor, like an apprenticeship, during periods when it’s only been about medicine. Being with a chiropractor, experiencing patient cases, and being privy to different types of patient management would be extremely useful and appreciated.* (Male, Year 4, 2012)

### A place of meaningfulness

This theme was interpreted within two categories: *Where it all happens* and *Detached worlds.*

During the clinical training, both in the outpatient clinic and the internship, the students experienced the tying of loose ends. This environment facilitated their experience of communal cohesion, giving them the opportunity to evolve, learn, and gradually become part of the profession. It was where theory was translated into practice and where factual knowledge assumed purpose and meaning. The students stated that the internship and outpatient clinic enabled them to understand the importance of taking responsibility for their own development and of identifying knowledge-, skill- and behavior-related gaps.

*It’s during the internship at the hospitals that you’re able to put theory into practice. You learn there and in the student outpatient clinic. It’s very good to have both […] different […] and very complementary.* (Female, Year 4, 2009)

The students experienced the outpatient clinic as a valuable, safeguarding training environment where the learning conditions permitted them to work autonomously, albeit under supervision, and where they had access to professional expertise. However, the quality of the environment was inextricably linked to the supervisor and the types and number of patients seen.

The students felt that the program was characterized by a continuum from systematic teaching in the first years to less systematic teaching in later years. They perceived the formal and classroom-based courses as more systematic than the clinical and practical courses. The latter were seen as more fragmentary, happenstance, and disorganized, and classroom-based teachers were more prepared for their sessions. The students experienced a sense of two worlds, receiving two diverse types of training in two diametric environments.

*…It’s become more fragmentary the more time we’ve spent here. It was more systematic in the beginning – I’d say in the first two to three preclinical years than in the last two clinical years. There is a stark difference between then and now; it’s like two different kinds of education.* (Male, Year 4, 2009)

There was a sense of a barrier between formal theoretical teaching inside the classroom and exogenously located clinical and practical teaching. The contrasting narratives of those students who were interviewed at the two time points revealed a deteriorative shift in the environment from organized to disconnected.

*During the first 3 years – the preclinical period – the curriculum was logical and systematic. Some courses overlapped, but there was a clear connection between them. However, during the last years – clinical training – the logical order disappeared, and the courses became less systematic.* (Male, Year 4, 2012)

### Trust in a regulated system

One category emerged in annotating this theme: *Operative organization and communication.*

Students expected reliance on a regulated system to entail organized settings, clear expectations, and responsiveness to change. Forward planning for what was expected and required of them was experienced as important in creating an environment with a sense of trust, security, and understanding of the curricula. Sudden organizational changes generated confusion and irritation, and students found it difficult to plan their studies, private lives, and external activities.

*The schedule must be laid down earlier and more rigidly so that there’s a sense of stability. Sudden changes are very disturbing and problematic. Many of us come from other cities/countries, and we can’t plan trips home because the schedule is not set or changes. For us poor students, this is difficult as ticket prices fluctuate.* (Male, Year 1, 2009)

Shortages in communication between the organization, students, and teachers led to anxiety and stress, creating rifts, engendering “them” and “us” conceptions, and creating feelings of helplessness and organizational uncertainty.

*It’s bad communication on the school’s part […] If there are problems with the schedule or placements, it would be great if they could give us prior notice of changes. Sometimes, it feels like the school organization and students inhabit two different worlds.* (Female, Year 4, 2012)

The students experienced the physical environment as creating a relaxed setting with a feeling of closeness and trust, providing opportunities to ask questions, discuss, and interact with teachers and peers. Maintaining and controlling the smallness of the institution were deemed important to promoting an environment for learning.

*We’re a small school. We have small classes, and everyone is pulling in the same direction. I think that’s great. It feels like the teachers use this smallness in their lessons in the big lecture halls; it’s very interactive, including when we meet in the small seminar rooms, because we help each other!* (Female, Year 4, 2012)

### Scaffolding relationships

This theme was interpreted within the category: *Establishing camaraderie and relations.*

The students valued the amicable atmosphere and the small size of the institution and saw it as a condition for bridging relationships. They saw these as very positive characteristics, fundamental to their well-being and the general overall feel of the atmosphere. Despite some communication dilemmas, the students felt that the institution enabled a comradely environment, providing for interactions between peers and faculty. They also considered that it provided a good social environment where everyone felt included and welcomed. Moreover, no one expressed discriminatory, bullying, or classist attitudes, neither from teachers nor peers.

*Friendships and relationships go beyond the class year, and you can spend time with anybody. You don’t meet everybody enough to establish deep contacts, of course, and it’s not like with the friends in the class. If you participate a lot in extracurricular activities, you can establish more meaningful contacts with teachers and people from other years.* (Female, Year 1, 2009)

The students encouraged one another to be part of the community to ensure that everyone was well and could keep up with the study pace. They considered that the training institution created a safe atmosphere, allowing strong social interactions, which engendered empathy and mutual trust and strengthened them as fellow humans, peers, and future professionals.

## Discussion

Using five themes, the analysis revealed a multilayered, cohesive educational environment that changes over time: *Personal growth*; *Being part of a community*; *A place of meaningfulness*; *Trust in a regulated system*; and *Scaffolding relationships*. These themes can be linked together and regarded as constituents of an educational environment.

According to the current literature, modern educational environments – as conditions for learning – should build on three elements: that learning occurs in a context, takes a constructivist approach, and takes place in collaboration [44]. However, our findings reveal some challenges in embedding training within such environments.

According to Moos [14], personal growth constitutes one of the key elements of human environments, having been described as encompassing the basic directions along which personal development and self-enhancement tend to occur in a particular environment. Our findings imply that pressure and demands and motivation and autonomy are counter poles, and teachers could mitigate pressure and demands by promoting autonomy, inspiring, and motivating as part of their pedagogical strategies. Kern et al. [45] postulate that outcomes of personal growth include changes in values and goals, improved relationships, and increased productivity and creativity. Thus, crafting educational environments that are apt for and incorporate personal growth is essential in creating necessary conditions for learning.

Our results infer that students had the desire to succeed in becoming part of a novel community. A core concept in the theory of communities of practice is the interface between novices and experts and the route whereby new members construct a professional identity [46]. Communities of practice encompass social arrangements in which individuals learn by sharing in activities and inclusiveness enables an understanding of norms and values and the ways in which the community functions and dysfunctions. Developing a professional identity is something personal, and social processes are not detached from the acquisition of knowledge, skills, and behaviors [47]; therefore, we argue that establishing pertinent environments that take communal belongingness into consideration is essential.

Most chiropractic training institutions are small and deliver the majority of their clinical training in campus-based outpatient clinics. One would imagine that remnants of isolated teaching and learning within silos would be easier to demolish in smaller institutions. Still, our findings reveal barriers between preclinical and clinical training and that meaningful learning is experienced mostly during the clinical training and internships. Wenger [37] asserts that our ability to experience the world and our engagement with it as meaningful is ultimately what learning should produce. Concurring with Shochet et al. [48], our premise is that in order to create environments that stimulate and embrace meaningfulness, the demolition of existing barriers could provide students with better learning opportunities that facilitate the development of knowledge that is relevant and meaningful, deep and retrievable, and amenable to alteration as part of an ongoing process.

In congruence with others [6, 49, 50], the students experienced organizational and communication problems with faculty, engendering feelings of stigmatization and a boundary between “them”, the training institution, and “us”, the students. Alienation and student discontent are not uncommon [51, 52], and the literature suggests they are increasing in universities [52, 53]. However, even though students expressed some problematic scheduling issues, faculty members might perceive these differently and often underline explicit course planning while students emphasize content, sequence, and co-ordination [54]. Moreover, while students are heterogeneous individuals, institutions’ educational strategies and teaching methods are usually homogeneous structures [55]. Thus, the notion of an all-embracing environment should incorporate a regulated system – in which students and teachers feel confident – as an important determinant of learning conditions.

In line with empirical findings [48, 56, 57], our findings suggest that social integration and student-teacher interaction created a scaffold for relationships, which was pertinent for the educational environment. Wenger [37] suggests that mutual engagement does not require sameness, but it does create relationships among people. When it is sustained, it bonds participants in ways that can become deeper than more elusive similarities in terms of personal features or social categories. Of this, a community of practice can grow into a very tight node of interpersonal relationships. The community of practice perspective on learning [37] – the act of becoming a member of a community of practice – foregrounds processes of relationship building: peer to peer and student to teacher.

Based on the findings, we argue that we should continue to create, develop, and refine educational environments that encourage and guide novices to become experts. Environments should engage and motivate students stepwise with increased complexity, in the context of safe and meaningful places and in relationships with other members of the community, and support them to become independent professionals.

### Limitations of the study

One potential limitation is that PJP was a teacher in the program, which may have discouraged the participants from speaking freely, potentially affecting the analysis. However, the year 1 participants were not assessed by the principal author during any of the examinations over the 2-year time frame of the study. For year 4 participants, this was not arbitrated as an issue as the investigator was not involved in teaching at this level. Consequently, any power imbalance in the interviews was reduced. The other investigator and our additional analyst were not involved in the program and contributed to outsiders’ views of the data. This analyst triangulation [38] can be seen as enhancing the credibility of the study. Another strategy for achieving credibility was frequent peer debriefing sessions [58]. Although focus groups provide a very effective forum for in-depth analyses of a topic, the sensitivity of certain aspects of the phenomenon may have thwarted this. Perhaps some issues of the environment are delicate, and some participants are unlikely to disclose personal experiences in group interviews. Semi-structured interviews could have proved a suitable alternative method. However, Wellings et al. [59] maintain that focus groups can indeed elicit responses and thoughts about sensitive topics and that focus group dynamics can provide data that are not generated by other methods. Confirmability was enhanced by documenting the content of all decision-making activities and discussions regarding emerging categories and themes. Stepwise replications enhanced the dependability of the analyses. Iterative intra- (among investigators) and inter-discussions (with peers) of the results and congruence on the credibility of the findings enabled further dependability of the analysis [42]. Reflexivity was present during the entire research process as the data from the study was continually discussed among the investigators.

Due to the small sample drawn from one metropolitan chiropractic training institution in Sweden, the findings may not be generalizable to other settings. While in-depth recollections of single and bounded training institutions are limited by their narrow focus, common themes can emerge from a range of solitary studies and lead to the development of theoretical generalizations. We also believe that our longitudinal component may have added stability to the data, which was not merely cross-sectional, thus enriching the findings. Furthermore, the explicit description of the contextual setting, the participants, and the analysis, together with the links drawn between the findings, the theory, and the prevailing literature, may create possibilities for the reader to appraise the transferability of the results.

Despite these empirical limitations, particularly the limited number of participants, this study contributes to a deeper understanding of educational environments in healthcare professional training.

### Implications for research

Empirical and theoretical evidence suggests that despite the intangibility and poorly understood nature of the concept of the educational environment, its effects are extensive, tangible, and persuasive. In concurrence with others [60], we argue that this phenomenon is not only due to perceptions of marginalized individuals but also to multidimensional factors with noticeable effects on educational outcomes. The results from this study can contribute to this line of thought. There is thus a need for further research on the factors and concepts involved. During the final analytical process of this study, we partially employed Moos’ [14] theory of human environments as a framework, as proposed by Schönrock et al. [11], to understand our findings. Some of our results align well with this conceptual model, but further empirical investigations are needed and would probably best be accomplished using multiple methods and different research paradigms.

We have asserted elsewhere that perceptions of the educational environment among student cohorts are idiosyncratic and may differ widely on a year-to-year basis [26, 61]. As teachers often remain in an educational environment for extended periods, it is plausible that they would perceive it differently. Scholars have alluded to the paucity of empirical studies investigating teachers’ perspectives [4, 5, 62, 63], pointing to the need for further research on how teachers perceive, conceive, and experience the environment as they are an intricate part of the environment perceived by students.

## Conclusions

This study advances new knowledge about chiropractic students’ experiences of the meaning of the educational environment and reveals some elusive factors contributing to a pertinent environment. Based on our findings we assert, the experience of the phenomenon under study changed over time. Early in the training, the educational environment was experienced as, *being part of a community* by establishing and understanding the vocational identity as well as *scaffolding relationships* through founding intra-institutional friendships and relations. In later stages, the environment was more about endorsing one’s *personal growth* – balancing academic pressures with professional progress and laying the foundation for autonomy and motivation. In the latter years, the clinical environment was experienced as where learning happens, thus creating *a place of meaningfulness*. Throughout the training, the formal and clinical environments were experienced as isolated, creating a feeling of detachment between trainings, with little bridging between the two. An environment incorporating stakeholders’ *trust in a regulated system* – an operative organization with clear communication channels regarding what to expect and with explicit rules and policies – was important for an apt education.

Our findings could be regarded as possible conditions for learning, and facets of the educational environment and could be taken into consideration when: restructuring or evaluating curriculums; interpreting results from quantitative measurements; constructing and refining instruments; and conceptualizing and framing the phenomenon.

## References

[1] Soemantri D, Herrera C, Riquelme A (2010). Measuring the educational environment in health professions studies: a systematic review. Med Teach.

[2] Dent J, Harden RM (2005). A practical guide for medical teachers.

[3] Isba R, Boor K. Creating a learning environment. In: Dornan T, Mann K, Scherpbier A, Spencer J, editors. Medical education: theory and practice. Edinburgh: Churchill Livingstone/Elsevier; 2011. p. 99–114.

[4] Genn JM (2001). AMEE Medical Education Guide No. 23 (Part 1). Curriculum, environment, climate, quality and change in medical education — a unifying perspective. Med Teach.

[5] Genn JM (2001). AMEE Medical Education Guide No. 23 (Part 2). Curriculum, environment, climate, quality and change in medical education — a unifying perspective. Med Teach.

[6] Till H (2004). Identifying the perceived weaknesses of a new curriculum by means of the Dundee Ready Education Environment Measure (DREEM) Inventory. Med Teach.

[7] Varma R, Tiyagi E, Gupta JK (2005). Determining the quality of educational climate across multiple undergraduate teaching sites using the DREEM inventory. BMC Med Educ.

[8] Edgren G, Haffling AC, Jakobsson U, McAleer S, Danielsen N (2010). Comparing the educational environment (as measured by DREEM) at two different stages of curriculum reform. Med Teach.

[9] Mulrooney A (2005). Development of an instrument to measure the practice vocational training environment in Ireland. Med Teach.

[10] Holt MC, Roff S (2004). Development and validation of the Anaesthetic Theatre Educational Environment Measure (ATEEM). Med Teach.

[11] Schönrock-Adema J, Bouwkamp-Timmer T, van Hell EA, Cohen-Schotanus J (2012). Key elements in assessing the educational environment: where is the theory?. Adv Health Sci Educ Theory Pract.

[12] Peterson MS, Spencer MG (1990). Understanding academic culture and climate. New Directions for Institutional Research.

[13] Pace CR, Stern GC (1958). An approach to the measurement of the psychological characteristics of learning environment. J Educ Psychol.

[14] Moos RH (1973). Conceptualizations of human environments. Am Psychol.

[15] Seabrook MA (2004). Clinical students’ initial reports of the educational climate in a single medical school. Med Educ.

[16] Hegenbarth M, Rawe S, Murray L, Arnaert A, Chambers-Evans J (2015). Establishing and maintaining the clinical learning environment for nursing students: a qualitative study. Nurse Educ Today.

[17] Liljedahl M, Engqvist Boman L, Porthén Fält C, Bolander Laksov K. What students really learn: contrasting medical and nursing students’ experiences of the clinical learning environment. Adv Health Sci Educ Theory Pract. 2014; doi10.1007/s10459-014-9564-y. 10.1007/s10459-014-9564-y 25312745

[18] Roff S, McAleer S, Harden RM, Al-Qahtani M, Ahmed AU, Deza H (1997). Development and Validation of the Dundee Ready Education Environment Measurement (DREEM). Med Teach.

[19] Boor K, Van Der Vleuten C, Teunissen P, Scherpbier A, Scheele F (2011). Development and analysis of D-RECT, an instrument measuring residents’ learning climate. Med Teach.

[20] Strand P, Sjöborg K, Stalmeijer R, Wichmann-Hansen G, Jakobsson U, Edgren G (2013). Development and psychometric evaluation of the Undergraduate Clinical Education Environment Measure. Med Teach.

[21] Whittle SR, Whelan B, Murdoch-Eaton DG (2007). DREEM and beyond: studies of the educational environment as a means for its enhancement. Educ Health (Abingdon).

[22] Denz-Penhey H, Murdoch JC (2009). A comparison between findings from the DREEM questionnaire and that from qualitative interviews. Med Teach.

[23] Colbert-Getz JM, Kim S, Goode VH, Shochet RB, Wright SM (2014). Assessing medical students’ and residents’ perceptions of the learning environment: exploring validity evidence for the interpretation of scores from existing tools. Acad Med.

[24] Snadden D (2006). Clinical education: context is everything. Med Educ.

[25] Mojaddidi MA, Khoshhal KI, Habib F, Shalaby S, El-Bab ME, Al-Zalabani AH (2013). Reassessment of the undergraduate educational environment in College of Medicine, Taibah University, Almadinah Almunawwarah, Saudi Arabia. Med Teach.

[26] Palmgren PJ, Sundberg T, Bolander Laksov K. Reassessing the educational environment among undergraduate students in a chiropractic training institution — a study over time. J Chiropr Educ. 2015 May 29. http://dx.doi.org/10.7899/JCE-14-37. 10.7899/JCE-14-37 PMC4582609 26023892

[27] McKendree J (2009). Can we create an equivalent educational experience on a two campus medical school?. Med Teach.

[28] Kohli V, Dhaliwal U (2013). Medical students’ perception of the educational environment in a medical college in India: a cross-sectional study using the Dundee Ready Education Environment questionnaire. J Educ Eval Health Prof.

[29] Ostapczuk MS, Hugger A, de Bruin J, Ritz-Timme S, Rotthoff T (2011). DREEM on, dentists! Students’ perceptions of the educational environment in a German dental school as measured by the Dundee Ready Education Environment Measure. Eur J Dent Educ.

[30] Foster Page LA, Kang M, Anderson V, Thomson WM (2012). Appraisal of the Dundee Ready Educational Environment Measure in the New Zealand dental educational environment. Eur J Dent Educ.

[31] Payne LK (2013). Comparison of students’ perceptions of educational environment in traditional vs. accelerated second degree BSN programs. Nurse Educ Today.

[32] Till H (2005). Climate studies: can students’ perceptions of the ideal educational environment be of use for institutional planning and resource utilization?. Med Teach.

[33] Palmgren PJ, Chandratilake M (2011). Perception of educational environment among undergraduate students in a chiropractic training institution. J Chiropr Educ.

[34] Creswell J (2013). Qualitative inquiry and research design.

[35] Watzlawick PB, Bavelas JB, Jackson DD (2011). Pragmatics of Human Communication: A Study of Interactional Patterns, Pathologies and Paradoxes.

[36] Swanwick T (2010). Understanding medical education—evidence, theory and practice.

[37] Wenger E (1988). Communities of practice: learning, meaning and identity.

[38] Patton M (2002). Qualitative research & evaluation methods.

[39] Krueger RA (1998). Developing questions for focus groups.

[40] Krueger R, Casey MA (2009). Focus groups: a practical guide for applied research.

[41] Kvale S, Brinkmann S (2009). Interviews. Learning the craft of qualitative research interviewing. 2nd ed.

[42] Graneheim UH, Lundman B (2004). Qualitative content analysis in nursing research: concepts, procedures and measures to achieve trustworthiness. Nurse Educ Today.

[43] Krippendorff K (2013). Content analysis: an introduction to its methodology 3rd ed.

[44] Hommes J, Rienties B, de Grave W, Bos G, Schuwirth L, Scherpbier A (2012). Visualising the invisible: a network approach to reveal the informal social side of student learning. Adv Health Sci Educ Theory Pract.

[45] Kern DE, Wright SM, Carrese JA, Lipkin M, Simmons JM, Novack DH, Kalet A, Frankel R (2001). Personal growth in medical faculty: a qualitative study. West J Med.

[46] Li LC, Grimshaw JM, Nielsen C, Judd M, Coyte PC, Graham ID (2009). Evolution of Wenger’s concept of community of practice. Implement Sci.

[47] Manninen K, Welin Henriksson E, Scheja M, Silén C (2013). Authenticity in learning – nursing students’ experiences at a clinical education ward. Health Educ.

[48] Shochet RB, Colbert-Getz JM, Levine RB, Wright SM (2013). Gauging events that influence students’ perceptions of the medical school learning environment: findings from one institution. Acad Med.

[49] Henning MA, Shulruf B, Hawken SJ, Pinnock R (2011). Changing the learning environment: the medical student voice. Clin Teach.

[50] Dunne F, McAleer S, Roff S (2006). Assessment of the undergraduate medical education environment in a large UK medical school. Health Educ J.

[51] Harrison TR (2007). My professor is so unfair: student attitudes and experiences of conflict with faculty. Conflict Resolution Quarterly.

[52] Bolkan S, Goodboy AK (2013). No complain, no gain: students’ organizational, relational, and personal reasons for withholding rhetorical dissent from their college instructors. Commun Educ.

[53] Cooper-Hind H, Taylor J (2012). Student complaints: an accurate measure of student dissatisfaction?. High Educ Rev.

[54] Muller Jain S, Loeser H, Irby DM (2008). Lessons learned about integrating a medical school curriculum: perceptions of students, faculty and curriculum leaders. Med Educ Online.

[55] Pyke SW (1996). Sexual harassment and sexual intimacy in learning environments. Can Psychol.

[56] Hoffman M, Morrow J, Salomone K (2002). Investigating “sense of belonging” in first-year college students. J Coll Stud Ret.

[57] Hutchinson L (2003). Educational environment. BMJ.

[58] Shenton AK (2004). Strategies for ensuring trustworthiness in qualitativeresearch projects. Educ Inf.

[59] Wellings K, Branigan P, Mitchell K (2000). Discomfort, discord and discontinuity as data: using focus groups to research sensitive topics. Cult Health Sex.

[60] Vaughan B, Mulcahy J, McLaughlin P (2014). The DREEM, Part 2: psychometric properties in an osteopathic student population. BMC Med Educ.

[61] Palmgren PJ, Lindquist I, Sundberg T, Nilsson GH, Bolander LK (2014). Exploring perceptions of the educational environment among undergraduate physiotherapy students. Int J Med Educ.

[62] Rotthoff T, Ostapczuk MS, De Bruin J, Decking U, Schneider M, Ritz-Timme S (2011). Assessing the learning environment of a faculty: psychometric validation of the German version of the Dundee Ready Education Environment Measure with students and teachers. Med Teach.

[63] Strand P, Edgren G, Borna P, Lindgren S, Wichmann-Hansen G, Stalmeijer RE (2015). Conceptions of how a learning or teaching curriculum, workplace culture and agency of individuals shape medical student learning and supervisory practices in the clinical workplace. Adv Health Sci Educ Theory Pract.

